# COVID-19 and the Endocrine System: A Review of the Current Information and Misinformation

**DOI:** 10.3390/idr14020023

**Published:** 2022-03-11

**Authors:** Samir Ahmed Mirza, Abdul Ahad Ehsan Sheikh, Michaela Barbera, Zainab Ijaz, Muhammad Ali Javaid, Rahul Shekhar, Suman Pal, Abu Baker Sheikh

**Affiliations:** 1 Department of Internal Medicine, Dow Medical College, Karachi 74200, Pakistan; samir.mirza1910@gmail.com (S.A.M.); muhammad.ali.javaid1995@gmail.com (M.A.J.); 2 Department of Internal Medicine, The Wright Center for Graduate Medical Education, Scranton, PA 18505, USA; abdulahad.esn@gmail.com; 3 Department of Internal Medicine, University of New Mexico, Albuquerque, NM 87106, USA; mbarbera@salud.unm.edu (M.B.); rshekhar@salud.unm.edu (R.S.); spal@salud.unm.edu (S.P.); 4 Department of Psychiatry, William Beaumont Hospital, Royal Oak, MI 48073, USA; zijaz@ualberta.ca

**Keywords:** coronavirus 2019, COVID-19, vaccine, endocrine, misinformation, diabetes, adrenal, pancreas, thyroid, fertility, hypothalamus, pituitary

## Abstract

Coronavirus disease 2019 (COVID-19) infection primarily involves the respiratory system but has many noteworthy extra pulmonary manifestations as well. We write this review to highlight the basis of some pathophysiological mechanisms of COVID-19 infection-induced endocrine dysfunction. Different scientific databases and institutional websites were searched to collect and consolidate the most up-to-date data relating to COVID-19 infection and endocrine systems. Hypopituitarism, central diabetes insipidus, SIADH, thyroid abnormalities, hyperglycemia, adrenal insufficiency, orchitis and alteration in sperm morphology have been reported in case reports of patients with COVID-19 infection. Data focusing on COVID-19 vaccination was also searched to summarize the effect, if any, on the endocrine system. Endocrinopathies noted post COVID-19 vaccination, including cases of adrenal hemorrhage, new onset Type II Diabetes Mellitus and subacute thyroiditis, are also discussed in this review. This review calls attention to the misinformation relating to COVID-19 vaccination with supposed endocrine effects such as infertility and problems with pregnancy. Rebutting these misconceptions can help increase compliance and maximize COVID-19 vaccination to the public.

## 1. Introduction

SARS-CoV-2 is a member of the Coronaviridae family, a group of enveloped, positive-sense, single-stranded RNA viruses. Many extrapulmonary manifestations of the coronavirus disease 2019 (COVID-19) have been reported involving the cardiovascular, renal, gastrointestinal and urinary systems. These widespread manifestations are attributed to the presence of the ACE2 (Angiotensin converting enzyme 2) receptor in these tissues, which is postulated to be at the center of the pathogenesis of COVID-19 [1]. Similarly, expression of the ACE2 receptor has also been reported in various endocrine tissues including the hypothalamus, pituitary, thyroid, gonads, and pancreatic islets [2]. Therefore, it is imperative to understand the way COVID-19 can alter the function of these tissues and cause pathology, especially considering the close interplay between various endocrine systems as part of the RAAS (renin–angiotensin–aldosterone system) pathway and the central role of ACE2 in this pathway.

During the last two years, a lot of new data has emerged with the aim to improve our knowledge pertaining to COVID-19, its manifestations and management. In this paper we review the current knowledge on endocrine manifestations of COVID-19. In addition, we present the up-to-date understanding of COVID-19 vaccination in relation to endocrinopathies. Many different cases of endocrine dysfunction have been reported in literature which are summarized in this review.

Additionally, optimal global COVID-19 vaccination has faced many threats and barriers, including but not limited to, vaccine inequity, financial constraints and misconceptions related to the vaccine. Some of the biggest misconceptions regarding the vaccine stems from misinformation such as vaccines causing ‘infertility’ or ‘pregnancy loss’. This poses a huge threat to public health and needs to be addressed to help maximize COVID-19 vaccination.

## 2. Materials and Methods

The authors searched PubMed/MEDLINE, Web of Science, CINAHL, Google scholar, and independent websites for free-text words and medical subject headings’ terms related to “coronavirus”, “SARS-CoV-2”, “COVID-19”, “hypopituitarism”, “cushing’s disease”, “cushing’s syndrome”, “growth hormone deficiency”, “diabetes insipidus”, “inappropriate antidiuretic hormone secretion syndrome”, “hyponatremia”, “hypernatremia”, “hypothyroidism”, “hyperthyroidism”, “thyroid nodule”, “thyroid carcinoma”, “hyperparathyroidism”, “hypoparathyroidism”, “hypercalcemia”, “hypocalcemia,” “vitamin D”, “hypercortisolism”, “addison’s disease”, ‘’pancreas’’, ‘’acute pancreatitis’’, “estrogen”, “testosterone”, “polycystic ovary syndrome”, “male hypogonadism”, “immune system”, “thrombosis”, ‘’COVID-19 vaccine’’, ‘’COVID-19 vaccine endocrinopathies’’, ‘’COVID-19 vaccine misconceptions’’, and ‘’COVID-19 misinformation’’. Case reports, case series, original studies, reviews, systematic reviews, and meta-analyses written in English were searched and selected, focusing on the following topics: pathophysiological mechanisms of endocrine dysfunction related to COVID-19 infection; relationship between endocrine dysfunction or possible side effects and COVID-19 vaccine; COVID-19 vaccine endocrinopathies; analysis of misinformation related to COVID-19 vaccination and endocrine systems.

### 2.1. COVID-19 and Endocrine System

**Figure 1:**
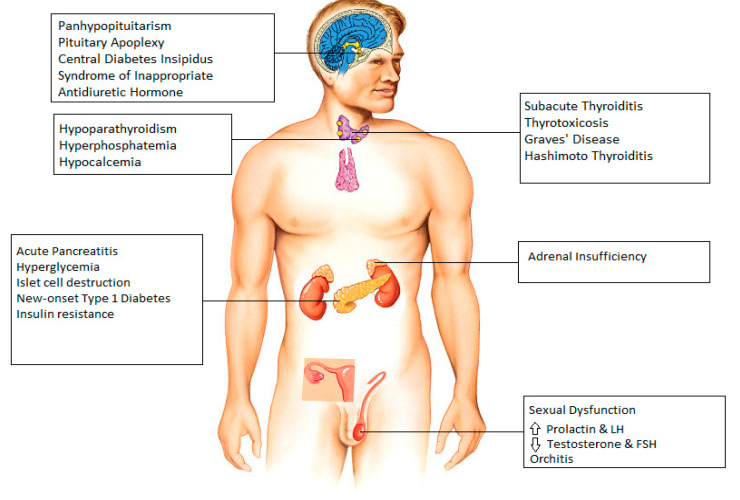
Endocrine manifestations seen after infection with COVID-19.

Molecular analysis of the SARS-CoV-2 has demonstrated the coronavirus spike (S) protein binds to host cells via ACE2 receptors and the S protein is subsequently cleaved by the serine protease TMPRSS2, an essential two-step process initiating the penetration and entry of the virus into the cell [3]. ACE2 receptors are abundantly expressed on respiratory tract and alveolar epithelial cells (type 2 pneumocytes, in particular), making the pulmonary system the primary target of COVID-19 [4]. Expression of the ACE2 receptor on endocrine tissues including the hypothalamus, pituitary, thyroid, gonads, and pancreatic islets has been studied. In our review, we tried to individually address different endocrine systems to better understand the acute, and in some cases chronic, effects of COVID-19 (Figure 1).

### 2.2. Hypothalamus and Pituitary

The expression of ACE2 in the hypothalamus was confirmed by Chigr et al., who identified the presence of ACE2 in the paraventricular nucleus, based on autopsy findings, making it a probable target for SARS-CoV-2 [5]. CT and MRI findings have revealed evidence of COVID-19 infecting the brain [6]. The SARS-CoV-2 genome has been detected in the cerebrospinal fluid of a patient with COVID-19, thereby confirming that SARS-CoV-2 does indeed infiltrate into the brain, and hence can involve any part of the brain, including the hypothalamus and pituitary [7].

There have been several case reports that indicate COVID-19-induced damage to hypothalamus and pituitary in different clinical scenarios. A case of a COVID-19-infected patient presenting with panhypopituitarism was detected in Turkey [8]. One study reports 10 cases of pituitary apoplexy who were found to have COVID-19 infection perioperatively [9]. Electrolyte abnormalities have frequently been reported in COVID-19 infection [10]. There are 2 separate case reports demonstrating improvement of hypernatremia in COVID-19 patients with desmopressin indicating central diabetes insipidus as the most likely underlying cause for patients’ presentations [11]. In contrast to this, a case report and a small series of three cases have reported the incidence of COVID-19 associated syndrome of inappropriate antidiuretic hormone resulting in hyponatremia [12,13].

### 2.3. Thyroid

Thyroid function abnormalities are a widely reported endocrine manifestation associated with COVID-19 infection. Evidence for the presence of ACE-2 receptors and TMPRSS2 in thyroid cells has been established [14,15]. Several cases of subacute thyroiditis have been reported, with symptoms typically occurring 16–36 days after the resolution of COVID-19 infection [16,17,18]. In a study by Lania et al., a significant number of patients (20.2%) hospitalized for COVID-19 were found to have thyrotoxicosis in absence of neck pain, likely identifying patients with COVID-19-related painless (silent/atypical) thyroiditis [19]. Whereas subacute thyroiditis is typically reversible and patients return to baseline with within few weeks of prednisone treatment, it is noteworthy to mention that COVID-19 patients with overt thyrotoxicosis are at increased risk of atrial fibrillation, thromboembolic events and higher in-hospital mortality and longer periods of hospitalization [19]. In addition, two cases of COVID-19-related Graves’ disease were documented by Mateu-Salat et al.; the authors concluded that COVID-19 could be a trigger for new cases or relapses of Graves’ disease [20].

Cases of COVID-19-related primary hypothyroidism have been reported in some studies [19,21]. A case of Hashimoto’s thyroiditis with subclinical hypothyroidism in a 45-year-old male and another case of myxedema coma in a 69-year-old woman have also been described [22,23]. Similar to overt thyrotoxicosis, hypothyroidism can negatively impact outcome of COVID-19 and is found to be associated with increased mortality [19].

Other common thyroid manifestations of COVID-19 include low-T3 syndrome; however, it is known that patients in the intensive care unit typically present with decreased triiodothyronine, low thyroxine, and normal range or slightly decreased TSH [24]. In addition, it should be taken into account that the evaluation of thyroid function in COVID-19 patients can be influenced by a number of medications [25]. Furthermore, abnormalities seen in thyroid function in patients with COVID-19 can also be attributed to disruption of the hypothalamic–pituitary–thyroid axis [26].

### 2.4. Parathyroid

Although there is limited data available describing parathyroid gland dysfunction related to COVID-19, tissue samples taken from deceased COVID-19 infected patients have identified SARS-CoV-2 RNA and antigenic materials in parathyroid gland acidophilic cells [27]. In addition, increased expression of angiotensin converting enzyme 2 (ACE2) receptors were detected in acidophilic cells of parathyroid glands, making the parathyroid gland a potential target for SARS-CoV-2 [28].

Elkattawy et al. reported a case of a 46-year-old patient admitted with severe COVID-19 infection who had hyperphosphatemia and low parathyroid hormone level; all possible causes of hypoparathyroidism had been excluded, which may indicate a COVID-19-related parathyroid dysfunction [29]. Interestingly a retrospective study by Liu et al. reported that two-thirds of patients with COVID-19 had hypocalcemia however, they described several mechanisms for this finding, including vitamin D deficiency, hypoalbuminemia, impaired intestinal absorption of calcium, hypoxic tissue damage with subsequent increase in calcium influx and impaired secretion of, and response to parathyroid hormone (PTH) secondary to increased levels of inflammatory cytokines, rather than singling out COVID-19-related direct parathyroid dysfunction as the possible cause [30].

### 2.5. Pancreas

Case reports and cohort studies have reported an association between COVID-19 and acute pancreatitis. ACE2 receptors are expressed in pancreatic ductal, acinar and islet cells [31]. In a cohort study of 121 COVID-19 patients, approximately 1% to 2% of non-severe and 17% of severe patients were found to have pancreatic injury [31]. A literature review revealed 22 cases of acute pancreatitis in patients with COVID-19 pneumonia, postulated causes included virus-mediated injury, systemic inflammatory response and circulating proinflammatory interleukins, virus-induced lipotoxicity, and drug-induced injury [32].

A study reported the incidence of hyperglycemia in COVID-19 patients to be as high as 50% [33]. Hyperglycemia is significantly associated with a prolonged ICU stay, higher need of mechanical ventilation, and increased risk of mortality in the critical care setting [34]. Hyperglycemia in patients with severe COVID-19 disease admitted to hospital may also be a result of the use of corticosteroids (Dexamethasone or equivalent) which is part of standard of care for severe COVID-19. Since hyperglycemia is also commonly seen in critically ill patients due to a cytokine storm, and ACE-2 receptors are also present on pancreatic islet cells, the underlying mechanism is a matter of controversy and is considered to be the combined result of both insulin resistance and insulin deficiency, secondary to islet cells destruction by the virus [34]. Hyperglycemia causes aberrant glycosylation of ACE2 receptors, which promotes the binding of the SARS-CoV-2 virus to it and thus increases the severity of COVID-19 disease, hence maintaining a strict glucose control in critically ill patients with COVID-19 remains of paramount importance [35,36]. A clinical trial to look for the emergence of new onset type 1 diabetes mellitus after COVID-19 is currently underway.

### 2.6. Adrenal Gland

ACE2 and TMPRSS2 are colocalized in adrenocortical cells [37]. A review of 5 clinical studies found that cortisol levels are lower in critically ill patients with COVID-19 as compared to those of non-COVID-19 critically ill patients [37]. Isolated incidence of adrenal insufficiency has been reported in several studies [38,39]. Sheikh et al. reported a case of central adrenal insufficiency with concurrent central diabetes insipidus in a patient with COVID-19 infection [40]. However, the true cause of hypocortisolism (primary vs. secondary to hypothalamic–pituitary dysfunction) remains a matter of debate. A prospective study evaluating serum cortisol and ACTH in patients with severe COVID-19 is presently underway, in the meantime it is suggested that the suspicion of adrenal insufficiency should be high be in COVID-19 infected patients who present with hyponatremia and hypotension refractory to vasopressors.

### 2.7. Gonads

The hypothalamic–pituitary–adrenal, thyroid, and gonad axes can all be rapidly affected by viral infections such as COVID-19, and impairment of it can lead to sexual dysfunction in males [41]. Although the testes are protected against external influences by the blood–testicular barrier, some viruses can pass through it and cause inflammation [42]. A study in men with COVID-19 reported high levels of prolactin and luteinizing hormone in contrast to low testosterone and follicle-stimulating hormone levels, reflecting primary testicular damage during active disease [43]. However, the underlying mechanism of this finding remains a matter of debate, postulated mechanisms including infection-induced oxidative stress, hypothalamic–pituitary axis dysfunction due to acute severe infection and direct gonadal damage [44,45]. More recent studies are addressing the question of long term spermatogenic failure and male infertility; however, data regarding these concerns is limited, to date [46]. Interestingly, a case report has described long term alterations in sperm DNA and morphology in a patient infected with COVID-19 [47]. Furthermore, SARS-CoV-2 has also been found in the semen of men with acute infection as well as in recovering patients [48]. In addition, a case of a COVID-19-positive 37-year-old male presenting with bilateral orchitis has been reported [49].

Although there is an abundance of ACE-2 receptors in the ovaries and oocytes, no information at present exists regarding possible ovarian dysfunction after COVID-19 infection or any long-term sequelae on female fertility [50,51].

### 2.8. Endocrinopathies and COVID-19 Vaccines

**Figure 2:**
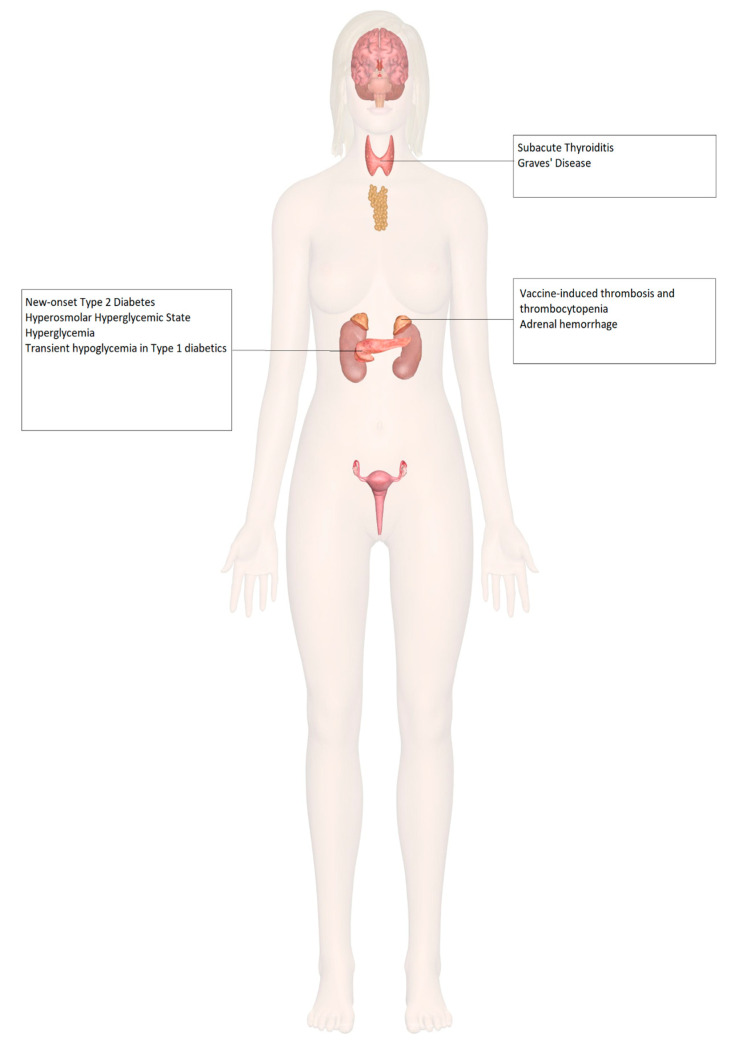
Endocrine organ involvement reported after COVID-19 vaccination.

COVID-19 vaccine trials, which included patients with endocrine disorders such as diabetes and obesity, demonstrated a similar efficacy and safety profile between endocrine patients and healthy volunteers without any pre-existing medical condition [48,52]. No differences in vaccine-associated adverse effects were reported in patients with endocrine disorders. Therefore, various clinical societies have issued guidance supporting COVID-19 vaccination of patients with stable endocrine disorders [53,54,55]. However, there have been a few reported cases of endocrinopathies post-vaccination, which are discussed in this review (Figure 2).

### 2.9. Adrenal Insufficiency

The use of corticosteroids is an integral part of the management of adrenal insufficiency. However, exogenous steroids can, in theory, alter the immune response induced by vaccines by transiently decreasing lymphocyte populations, causing apoptosis of T lymphocytes, and altered secretion of immunoglobulins [56,57]. However, these effects and subsequent infections are primarily seen in patients who are prescribed daily prednisone doses above 10 mg; therefore, for most patients, no change in vaccination protocol is needed [58].

In patients with adrenal insufficiency, it is not recommended to pre-emptively increase the glucocorticoid dose [55], nor is it recommended to discontinue glucocorticoid treatment before COVID-19 vaccination, considering that prednisolone up to 20 mg/day did not suppress the immune system in patients with adrenal insufficiency being vaccinated against influenza [59]. Despite the impairment in vaccine-based immunity, the effect on efficacy has been minute [60,61].

Increased incidences of prothrombotic states associated with thrombocytopenia have been seen with certain COVID-19 vaccines, such as the ChAdOx1 (AstraZeneca, University of Oxford) and the Ad26.COV2.S (Janssen; Johnson & Johnson) vaccines. There has been a documented case of vaccine-induced thrombosis and thrombocytopenia with bilateral adrenal hemorrhage [62], as well as a case of left adrenal hemorrhage [63] following COVID-19 vaccination, both described in Table 1.

### 2.10. Diabetes Mellitus

Though there is a lack of literature pertaining to the immune response to COVID-19 vaccination among diabetics, one recent study showed the antibody response against multiple severe acute respiratory syndrome coronavirus 2 (SARS-CoV-2) antigens in patients with diabetes, in terms of timing and titers, had no difference compared to that of individuals without diabetes, and was not found to be influenced by glucose levels [64]. Further effects can be extrapolated from clinical evidence of immune responses to hepatitis B, pneumococcal, and influenza vaccines in diabetic patients [65,66,67,68], which are recommended as standard of care among these patients and have been shown to be efficacious and safe.

There has been a documented case of new-onset type 2 diabetes mellitus presenting as the hyperosmolar hyperglycemic state (HHS) [69], 3 cases of fasting and postprandial hyperglycemia in patients with well-controlled type 2 diabetes mellitus [70], and transient hypoglycemia in patients with type 1 diabetes mellitus on basal-bolus insulin regimens after COVID-19 vaccination [71], described in Table 1.

### 2.11. Thyroid Disorders

According to the American Thyroid Association and European Thyroid Association, medically stable patients with thyroid disorders, such as autoimmune thyroid disease and thyroid cancer, should receive COVID-19 vaccination. There is no evidence of increased risk of vaccination-associated infection adverse effects among patients with thyroid disease. However, rare cases of thyroid disorders have been documented following COVID-19 vaccination.

Three cases of subacute thyroiditis among young female healthcare workers have been reported following inactivated SARS-CoV-2 vaccine (CoronaVac) [72]. Interestingly, two of the three patients were actively breastfeeding, raising concern for the postpartum period as being a risk factor for developing a hyperimmune response to adjuvants or inactivated viral particles in the vaccine, resulting in the presentation of subacute thyroiditis. Three other cases of subacute thyroiditis have also been documented after vaccination [73,74,75]. The possible underlying mechanism might be attributed to post-vaccination autoimmune/inflammatory syndrome induced by adjuvants (ASIA syndrome) [76]. In addition, two female health care workers were diagnosed with Graves’ disease based on low TSH levels, elevated thyroid hormone levels, and elevated anti-thyroid antibodies following COVID-19 vaccination [77]. All these cases are further described in Table 1.

### 2.12. Hypogonadism and Infertility

Females have been observed to have a more robust immune response leading to better protection against the infection [78]. However, this strong immune response in females is accompanied by increased vaccination-related adverse effects, including the disproportionate incidence of anaphylaxis among female patients after administration of the Pfizer/BioNTech and Moderna COVID-19 mRNA vaccines [79]. Testosterone has been shown to decrease immune responses to vaccines [80,81,82]. Currently, there are no documented cases of hypogonadism induced by COVID-19 vaccination or data to suggest that vaccination can cause infertility.

### 2.13. Osteoporosis

As osteoporotic patients tend to be from an older demographic, their vaccination should be prioritized, which can, in turn, result in increased levels of outdoor activity and decreased rates of bone loss. Some osteoporotic medications may be needed, to be used with caution, while receiving COVID-19 vaccination. For example, intravenous bisphosphonates are known to cause an acute phase reaction of fever, flu-like symptoms, and myalgia. Hence, it is recommended their administration be separated from COVID-19 vaccination by a 1-week interval. An interval of 4–7 days is also recommended between the administration of denosumab and romosozumab and COVID-19 vaccination, due to potential injection site reactions, such as localized pain, swelling, and/or erythema [83].

No cases of accelerated bone loss or osteoporotic fractures have been reported post-vaccination with continued osteoporosis therapy.

### 2.14. Misinformation: COVID-19 Vaccine and Endocrine System

Misinformation about COVID-19 vaccination is a major threat to public health. Studies have shown a clear link between susceptibility to misinformation and compliance with public health guidance measures [84]. Therefore, to optimize COVID-19 vaccination, the government and trusted health care providers need to develop strategies not only to identify and address common misconceptions but also provide sources of fact-checked information for public access.

The endocrine system has been subject to some of the most popular misconceptions regarding COVID-19 vaccination. In a survey of a Middle Eastern population, approximately 60% believed the virus was man-made, and 23.4% believed vaccination would result in infertility; unsurprisingly, belief in such theories was more common among respondents getting information from social media as compared to medical professionals and scientific journals [85]. In the United States, Google searches for the phrase “infertility AND COVID vaccine” increased by 34,900% between February 2020 and February 2021 [86]. Infertility and effects on pregnancy were some of the most frequently asked questions in a survey of staff members of various skilled nursing facilities, demonstrating the increased concern of these perceived outcomes in the absence of any concrete data [87].

In fact, a recent prospective study comparing sperm parameters in 45 males before and after a median of 75 days post-vaccination, published by Gonzalez and colleagues at the University of Miami, showed a significant increase in semen volume, sperm concentration, sperm motility, and total motile sperm count compared to baseline values. In addition, sperm counts in 7 out of 8 men who were previously oligospermic before vaccination remarkably increased to normal levels [88]. In another study by Lifshitz, semen samples from 75 fertile men were analyzed after their COVID-19 vaccine. After an average of 37 days from the second dose of the vaccine, semen parameters were noted to be within reference range proving no detrimental effect from the vaccine [89].

There is also misleading information regarding the effect of COVID-19 vaccines in pregnancy, for example, that they may cause pregnancy loss. However, according to Dr. Anthony Fauci, 20,000 pregnant women have been vaccinated against COVID-19 in the United States as of March 2021 without any major adverse effects [90]. Moreover, there is evidence that pregnant women who contract COVID-19 are at higher risk of complications such as stillbirths, preterm labor, and death in the postpartum period [91,92].

In a recent report issued in January 2022 by CDC, a retrospective cohort of more than 40,000 pregnant women was studied, of which 10,064 pregnant women received at least 1 dose of COVID-19 vaccine during pregnancy. The report showed no association with preterm birth or small-for-gestational-age (SGA) at birth [93]. In another study, conducted at St George’s University Hospitals in London, 1328 pregnant women were studied, of whom 140 received at least 1 dose of COVID-19 vaccine. No adverse pregnancy outcomes were noted in vaccinated women as compared to unvaccinated [94]. On the other hand, women with symptomatic COVID-19 during pregnancy have been noted to be at a significantly higher risk for intensive care unit admission, need for mechanical ventilation, and a 70% increased risk for death, compared with nonpregnant women with COVID-19 infections [95]. Hence, the benefits of immunization in pregnant females outweigh the risks of serious complications of the virus during pregnancy. Table 2 summarizes all the studies addressing the misinformation related to COVID-19 vaccine and endocrine system.

**Table 1:** Reported endocrinopathies in patients post-COVID-19 vaccination.

Author (Country, Year)	System Involved	Age and Sex of the Patient	Type of Vaccine	Onset of Symptom	Presenting Symptoms	Final Diagnosis	Complications	Treatment	Outcome
Taylor et al. (Wales, 2021) [71]	Adrenal	38, Male	Astra Zeneca	8 days after Dose 1	Severe abdominal pain Vomiting	Vaccine–Induced Thrombosis and Thrombocytopenia with Bilateral Adrenal Hemorrhage	Dural venous sinus thrombosis	Intravenous Immunoglobulin, Hydrocortisone, Argatroban Plasma exchange	Improved platelet count after plasma exchange
Boyle et al. (United Kingdom, 2021) [72]	Adrenal	55, Female	Astra Zeneca	8 days after Dose 1	Left iliac fossa pain Vomiting	Left Adrenal Hemorrhage	Thrombo-embolism in both lungs, left basilic vein, and left renal vein	Hydrocortisone Apixaban	Positive response to therapy, conservatively managed further
Abu-Rumaileh et al. (Jordan, 2021) [78]	Diabetes	58, Male	Pfizer/BioNTech	21 days after Dose 1 (2 days after Dose 2)	Nocturia Polyuria Polydipsia Altered mental status Weight loss	Hyperosmolar Hyperglycemic State		IV Fluids Insulin drip Glargine 50 units daily plus 10 units pre-meal insulin	Insulin tapered and stopped in 4 weeks Metformin continued with good glycemic control
Mishra et al. (India, 2021) [79]	Diabetes	58, Female	Covishield	1 day	None Hypertension and tachycardia None	Exacerbation of hyperglycemia in pre-existing Type 2 Diabetes Mellitus	None	Increased dose of Metformin in patient 1 No interventions in patients 2 and 3	Return to previous blood glucose levels in 1 month, 3 days and 15 days, respectively
		64, Male							
				1 day					
		65, Male		6 days					
Heald et al. (United Kingdom, 2021) [80]	Diabetes	20 patients Median age 53 (range 26–70), 11 Females, 9 Males	Pfizer/BioNTech [8] Astra Zeneca [12]	7 days	None	Transient hypoglycemia in Type 1Diabetes Mellitus patients	None	No intervention	Return to previous glucose levels in further 7 days
Irlemi et al. (Turkey, 2021) [81]	Thyroid	35, Female	CoronaVac	4 days after Dose 2 4 days after Dose 1 7 days after Dose 2	Anterior neck pain Fever Palpitations Weight loss Fatigue	Subacute Thyroiditis (secondary to ASIA syndrome)	Recurrent myalgia and neck pain in patient 2	Methylprednisolone 16 mg once daily propranolol 25 mg twice daily No intervention in patient 3	Complete resolution of symptoms
		34, Female							
		37, Female							
Franquemont et al. (USA, 2021) [82]	Thyroid	42, Female	Pfizer/BioNTech	5 days after Dose 1	Sore throat Palpitation Tachycardia	Subacute Thyroiditis	None	Prednisone 40mg daily and Propranolol 20mg as needed	Rapid improvement of symptoms after therapy
Oyibo (United Kingdom, 2021) [83]	Thyroid	55, Female	Astra Zeneca	21 days after Dose 1	Neck pain Swelling Headache Sore throat Myalgia Palpitation	Subacute Thyroiditis	None	Levothyroxine 50 mg daily Propranolol	Resolution of symptoms after therapy
Sahin et al. (Turkey, 2021) [84]	Thyroid	67, Male				Subacute Thyroiditis			
Vera-Lastra et al. (Mexico, 2021) [86]	Thyroid			3 days		Grave’s disease			

**Table 2:** Summary of studies addressing misinformation about COVID-19 vaccines and endocrine system.

Author	Study Design	Criteria	Patient Population	Conclusion	Limitations
Gonzalez et al. [88].	Single-center prospective study	Inclusion: Men aged 18–50, no underlying fertility issues.	45 male participants.	No significant decrease in sperm parameters after 2 doses of COVID-19 vaccination.	Small number of cohorts. Lack of control group.
		Exclusion: COVID-19 symptoms or positive results within the last 90 days.			
Lifshitz et al. [89].	Prospective cohort study	Inclusion: Men < 45 years old, fertile men were considered to be those who had previously successfully impregnated their partners without the use of artificial reproductive technology.	75 male participants.	Semen parameters found to be within normal parameters after COVID-19 vaccination.	Participants from the same socioeconomic group. Only tested once after being vaccinated and not before the vaccination. Potential long-term effects not tested.
		Exclusion: Previously diagnosed with SARS-CoV-2 infection, taking medications known to be detrimental to semen parameters.			
Lipkind et al. [93]	Retrospective cohort study	Inclusion: Single-gestation pregnancies.	46,079 participants.	COVID-19 vaccination during pregnancy was not significantly associated with increased risk for preterm birth overall or SGA at birth	Some vaccinations might have been missed due to data from multiple resources. Data on confounders were not available. Date on previous COVID-19 infection not available. Vaccinations not stratified according to trimesters.
		Exclusion: Age < 16 or >49 years, multiple gestations, no documented care in the health system, implausible gestational age, pregnancy start date outside the prespecified periods.			
Blakeway et al. [94].	Retrospective cohort study	Inclusion: Pregnant women with known vaccination status, complete maternal and fetal outcome data.	1328 Participants.	Similar pregnancy outcomes seen in vaccinated and unvaccinated participants.	Median time to birth after vaccination was just one month. Not vaccinated in the first trimester. Low power of the study so small or very small differences may have been missed.
		Exclusion: Complicated pregnancies with genetic syndromes, fully vaccinated before getting pregnant.			

## 3. Conclusions

Several endocrinopathies have been reported in patients with a COVID-19 infection. These include hypopituitarism, SIADH, central diabetes insipidus, thyroiditis, thyrotoxicosis, hypothyroidism, low T3 syndrome, hyperglycemia, adrenal insufficiency, orchitis, and alterations in sperm morphology. Most of the data is reported from isolated case reports and case series, and larger cohort studies would be needed to confirm these associations.

Endocrine abnormalities after COVID-19 vaccination include one case of vaccine associated thrombosis and thrombocytopenia with adrenal hemorrhage, a few cases of derangements in glycemic control including one case of new onset type 2 diabetes mellitus, and some cases of subacute thyroiditis.

Misinformation about COVID-19 vaccination, such as infertility in men and adverse effects on pregnancy in women, are not substantiated by the data available.

## Data Availability

Not applicable.
